# Dexmedetomidine for Alcohol Withdrawal: Looks Can Be Deceiving

**DOI:** 10.7759/cureus.95851

**Published:** 2025-10-31

**Authors:** Morgan Riggan, Zachary Schmitz, Robert S Hoffman, Nabil Biary, Rana Biary

**Affiliations:** 1 Department of Medicine, Division of Emergency Medicine, Western University Schulich School of Medicine and Dentistry, London, CAN; 2 Department of Emergency Medicine, New York Presbyteriain Brooklyn Methodist Hospital, Brooklyn, USA; 3 Division of Medical Toxicology, Ronald O. Perelman Department of Emergency Medicine, New York University (NYU) Grossman School of Medicine, New York City, USA; 4 Department of Neurology, KabaFusion, Lexington, USA

**Keywords:** alcohol withdrawal, critical care, patient-reported experience, seizure, dexmedetomidine

## Abstract

Alcohol is a gamma-aminobutyric acid (GABA) receptor agonist and an N-methyl-D-aspartate (NMDA) receptor antagonist. Although benzodiazepines and barbiturates are the standard treatments for alcohol withdrawal, there is some recent interest in adding dexmedetomidine. We report cases of two patients with severe alcohol withdrawal to highlight some limitations of dexmedetomidine therapy. The first case was of a 58-year-old man who presented with severe alcohol withdrawal. He received chlordiazepoxide, diazepam, and phenobarbital with resolution of his symptoms. He was later started on dexmedetomidine (0.2 mcg/kg/hr), with no other therapy, during which time he appeared sedated with a heart rate (HR) of 80 beats/minute, blood pressure (BP) of 126/80 mmHg, and respiratory rate (RR) of 18 breaths/minute. Seven hours after stopping the infusion, he said that he “had been lying in bed staring at the ceiling feeling like hell… felt extremely agitated in my head". The second patient was a 41-year-old man who developed alcohol withdrawal and required intubation seven hours after presentation. Maximal sedation was 50 mcg/kg/min of propofol and 14 mg/hr of midazolam. On hospital day 3, the team stopped propofol, decreased midazolam to 10 mg/hour, and added dexmedetomidine 0.2 mg/kg/hr. Forty minutes later, the toxicology team observed the patient unresponsive with left gaze deviation and rhythmic eye movements. Video EEG was consistent with a seizure. The patient’s EEG rapidly improved with a midazolam bolus.

Although dexmedetomidine controls the autonomic instability and behavior associated with alcohol withdrawal, it does not address the underlying pathophysiology, leaving patients to suffer the psychological effects of withdrawal. The role of dexmedetomidine remains to be determined, with studies that address cognitive and long-term outcomes of alcohol withdrawal rather than just vital signs, behavior, and doses of other medications used.

## Introduction

It is estimated that 14% of adults suffer from alcohol use disorder (AUD) worldwide [1]. Among inpatients, the incidence of alcohol withdrawal is between 2% and 7% [2]. Risk factors for the development of severe alcohol withdrawal include a previous history of delirium tremens, having more than one seizure during their current withdrawal episode, and prolonged heavy use of alcohol [2, 3].

Alcohol is a central nervous system (CNS) depressant. It increases inhibitory tone by enhancing the effects of gamma-aminobutyric acid (GABA) at GABA-A receptors and reduces excitatory tone through disruption of glutamatergic neurotransmission at the N-methyl-D-aspartate (NMDA) receptor [4, 5]. Chronic alcohol use results in adaptive processes in the central nervous system to maintain homeostasis. These include downregulation and desensitization of GABA-A receptors and upregulation of NMDA receptors [4, 5]. The sudden withdrawal of alcohol leaves the patient in a state of enhanced NMDA receptor expression and down-regulated GABA receptor expression, resulting in an excess excitatory state [4]. 

The manifestations of alcohol withdrawal are broad. Mild alcohol withdrawal can present with irritability, insomnia, tremulousness, palpitations, and diaphoresis. As the severity increases, autonomic instability, hallucinosis, and seizures occur, and in its most severe form, delirium tremens. Delirium tremens is a life-threatening condition of severe autonomic dysfunction leading to tachycardia, hypertension, and hyperthermia, as well as agitation and hallucinations. Mortality for untreated delirium tremens, which was once reported to be as high as 51% [6], is now reported to be between 0% and 7% [7-9]. Patients with delirium tremens still require prolonged ICU stays and have a high risk for aspiration, pneumonia, and the need for endotracheal intubation [9]. 

The treatment of patients with alcohol withdrawal is directed at addressing the underlying pathophysiology of the disease. Benzodiazepines, barbiturates, and propofol are all therapies with proven safety and efficacy in alcohol withdrawal [5, 10-13]. Dexmedetomidine has recently been proposed as a benzodiazepine-sparing treatment for acute alcohol withdrawal. Previous studies have evaluated the effects of dexmedetomidine in alcohol withdrawal patients, looking at the total amount of benzodiazepines given, the need for mechanical ventilation, intensive care unit (ICU) length of stay, and changes in heart rate and blood pressure [14-17]. Unfortunately, none of these are patient-centered outcomes. We report two cases of severe benzodiazepine-refractory alcohol withdrawal treated with dexmedetomidine to highlight the unpleasant patient experience during this therapy as well as to demonstrate that dexmedetomidine does not prevent alcohol withdrawal seizures.

## Case presentation

Case 1

A 58-year-old man with a history of AUD and prior ICU admissions for delirium tremens presented to the emergency department in alcohol withdrawal. His last drink was the night before presentation. On presentation to the emergency department, his vital signs were heart rate (HR), 100 beats/minute; blood pressure (BP), 160/90 mmHg; respiratory rate (RR), 18 breaths/minute; oxygen saturation, 97% on room air; afebrile; and normoglycemic. He had tremors in his extremities and tongue, but no hallucinations. He was given oral chlordiazepoxide and admitted under the internal medicine service for further management of his withdrawal. Over the next 48 hours, he received a total of 1150 mg chlordiazepoxide orally and 560 mg diazepam IV but remained symptomatic (Table 1).

**Table 1:** Case 1: treatments administered during hospital stay IV: intravenous; IM: intramuscular; PO: per oral; DEX: dexmedetomidine

Admission Day	Total Daily Treatments
1	Chlordiazepoxide 350 mg PO
	Diazepam 20 mg IV
	Haloperidol 2 mg IM
2	Chlordiazepoxide 300 mg PO
	Diazepam 200 mg IV
3	Chlordiazepoxide 500 mg PO
	Diazepam 340 mg IV
	Phenobarbital 65 mg IV
	Haloperidol 2 mg IV
4	Chlordiazepoxide 200 mg PO
	Diazepam 220 mg IV
5	Chlordiazepoxide 275 mg PO
	Haloperidol 1 mg IM
	Lorazepam 2mg IM
	DEX started at 10 AM (0.2 mcg/kg/hr)
6	DEX stopped at 5 AM (0.2 mcg/kg/hr)
	Chlordiazepoxide 375 mg PO
	Diazepam 180 mg IV
	Haloperidol 0.4 mg IV
	Haloperidol 5 mg IM
	Lorazepam 4 mg IM
	Lorazepam 4 mg PO
7	Chlordiazepoxide 300 mg PO
	Diazepam 140 mg IV
8	Chlordiazepoxide 450 mg PO
9	Chlordiazepoxide 225 mg PO
	Olanzapine 2.5 mg IM
10	Chlordiazepoxide 150 mg PO
	Olanzapine 2.5 mg PO

At this point, he was classified as benzodiazepine-resistant alcohol withdrawal and transferred to the ICU for further care. The medical toxicology service did a bedside assessment and recommended switching to phenobarbital. He was subsequently given 65 mg IV phenobarbital. Within 30 minutes, his vital signs normalized, and he had a Richmond Agitation-Sedation Scale (RASS) score of -2. Despite the medical toxicology recommendation and improvement with phenobarbital, it was decided by the bedside team to continue with benzodiazepines. Over the next 36 hours, his withdrawal symptoms began to progress, and he received a total of 475 mg chlordiazepoxide, 220 mg diazepam IV, 2 mg lorazepam IM, and 1 mg haloperidol IM without improvement. He developed severe agitation and autonomic instability. No further phenobarbital was given. Psychiatry was consulted and recommended dexmedetomidine, and he was started on an infusion of dexmedetomidine (0.2 mcg/kg/hr) as the sole therapy. During this time, he was assessed at the bedside by the medical toxicology service. He had a RASS score of -4, and his vitals were HR, 80 beats/min; BP, 126/80 mmHg; RR, 18 breaths/min; and afebrile. He was not interviewable while on the dexmedetomidine infusion. After 19 hours of dexmedetomidine, his infusion was stopped. Seven hours after stopping the dexmedetomidine, he was once again in moderate alcohol withdrawal. The medical toxicology service was again consulted, and another bedside assessment was made. When interviewed by the medical toxicology service, he stated that over the previous 24 hours, while on the dexmedetomidine infusion, he “had been lying in bed staring at the ceiling feeling like hell”. He said he “felt extremely agitated in my head” during that time period. He was restarted on symptom-triggered benzodiazepine dosing, and his severe alcohol withdrawal resolved over the following week. 

Case 2

A 41-year-old man presented to the emergency department with a decreased level of consciousness due to alcohol intoxication. After waking, he developed a tremor and tachycardia to 130/min and was given 100 mg of oral chlordiazepoxide. Two hours later, he was so tremulous that he could not walk. Ethanol concentration at that time was 332 mg/dL (72 mmol/L). His agitation worsened despite a total of 110 mg of IV diazepam and 260 mg of IV phenobarbital, and he required intubation seven hours after presentation. He was placed on 50 mcg/kg/min of propofol and 14 mg/h of midazolam and admitted to the ICU. On hospital day 3, the ICU team stopped propofol, decreased midazolam to 10 mg/h, and added dexmedetomidine 0.2 mcg/kg/h. Forty minutes later, the rounding toxicology team observed the patient unresponsive with left gaze deviation and rhythmic eye movements. Video EEG was consistent with a seizure. The patient’s clinical status and EEG rapidly improved with a midazolam bolus. 

## Discussion

The primary therapy for patients with alcohol withdrawal includes medications to restore inhibitory tone. The best evidence is for benzodiazepines and barbiturates, both of which stimulate the GABA receptor to counter the enhanced excitatory tone. Much research has shown that symptom-triggered therapy with benzodiazepines leads to less severe withdrawal symptoms, lower rates of delirium tremens, and shorter hospital lengths of stay [18-20].

Although there is no standard definition of benzodiazepine-resistant alcohol withdrawal [21], barbiturates are effective for those who fail to respond to benzodiazepines. Gold et al. showed that in patients admitted to the ICU for delirium tremens, escalating doses of diazepam with barbiturates reduced the need for mechanical ventilation and ICU length of stay [10]. Barbiturates augment the efficacy of benzodiazepines at the GABA receptor. The result of these therapies is a resolution of CNS excitation manifested by a calm, adequately sedated patient. The restoration of inhibitory tone in the CNS leads to improvement in heart rate, blood pressure, and temperature. 

Central α2-adrenergic receptor agonists can improve vital signs and lead to sedation, but do not address the underlying pathophysiology. Clonidine has previously been studied for use in alcohol withdrawal and has been shown to be ineffective, leading to more adverse events when compared to chlormethiazole [22]. Dexmedetomidine is the dextro-enantiomer of medetomidine. It acts as a selective α2-adrenergic receptor agonist with an α2/α1 selectivity ratio more than seven times greater compared to clonidine [23]. It also acts as an imidazoline receptor agonist [24]. Further, it has no affinity for the beta1, beta2, histamine-1, histamine-2, 5-HT1, 5-HT2, mu-type and delta-type opioid, dopamine-2, GABA, and benzodiazepine receptors [23]. There are three different α2-adrenergic receptor subtypes: 2A, 2B, and 2C. Pre-synaptic central α2A receptor agonism inhibits the release of norepinephrine from the vasoregulatory region of the CNS, specifically the locus ceruleus, the nucleus tractus solitarii in the medulla, and the rostral ventrolateral medulla, whereas post-synaptic stimulation of central α2 receptors inhibits sympathetic activity [25, 26]. This results in sedation, anxiolysis, bradycardia, and hypotension. Central agonism of the imidazoline receptors also decreases sympathetic outflow from the intermediolateral cell columns of the thoracolumbar spinal tracts to peripheral neurons, resulting in a reduction in heart rate and blood pressure [24, 25]. Outwardly, the use of this medication results in what appears to be a calm, sedate patient with normal vital signs. However, since there is no GABAergic property to this medication, it lacks the primary property needed to adequately address the pathophysiology of alcohol withdrawal: reversal of the loss of inhibitory tone. 

Previous studies have shown that dexmedetomidine reduces benzodiazepine requirements in the ICU and decreases ICU length of stay [14, 15, 17, 27, 28]. A randomized controlled trial of a dexmedetomidine infusion versus diazepam boluses concluded that dexmedetomidine results in lower 24-hour and cumulative benzodiazepine needs. However, in this patient population, the diazepam doses were small-20 mg in the dexmedetomidine group over 24 hours and 40 mg in the control group [28]. These doses are hardly suggestive of significant alcohol withdrawal or that the addition of dexmedetomidine had any meaningful reduction in benzodiazepine use. Other studies have found no difference in benzodiazepine use when adjusted for withdrawal severity or only found a significant decrease in benzodiazepines for the first 24 hours of therapy, with no difference in benzodiazepine requirements over seven days [15, 16]. 

Using benzodiazepine requirements as a poor surrogate marker as an endpoint for efficacy in the management of alcohol withdrawal. It is expected that dexmedetomidine would improve peripheral markers of withdrawal, such as tachycardia and hypertension, resulting in lower Clinical Institute Withdrawal Assessment (CIWA) scores and less benzodiazepine use. However, the total benzodiazepine dose used is not a patient-centered outcome. The vital signs are not reflective of what is happening on a cellular level in the central nervous system. We cannot use the resolution of tachycardia and hypertension with dexmedetomidine as proof that the increased excitatory tone and decreased inhibitory tone underlying alcohol withdrawal have been corrected, the ultimate goal in the management of alcohol withdrawal. Patients may still be experiencing significant distress despite improvement in vital signs and apparent sedation, as our first case highlights. Utilizing the RASS score alone as an endpoint is not an appropriate measure for the effectiveness of therapy in the management of alcohol withdrawal. One must also consider the underlying pathophysiology of the disease and provide therapy that targets this. Just because a patient no longer manifests vital sign abnormalities and is unresponsive does not mean that the withdrawal is treated. This is the equivalent of documenting improved pain in a patient who received morphine for a surgical abdomen and then deciding not to take the patient to the operating room. 

Dexmedetomidine is not without risks. When used as an adjunct therapy, dexmedetomidine leads to more bradycardia [14, 16, 28-30]. One case series describes a patient who developed two asystolic pauses of nine seconds each that were attributed to the dexmedetomidine infusion being used for alcohol withdrawal [17]. The use of dexmedetomidine as an adjunct reduced the rates of intubation in some studies [14], but not others [27, 29], and resulted in an increased need for intubation when used with phenobarbital [31]. A recent systematic review and meta-analysis on the use of dexmedetomidine for alcohol withdrawal found that it does not decrease the rate of endotracheal intubation and increases the risk of bradycardia [32].

Another limitation of studies that compare dexmedetomidine to benzodiazepines is the use of adjuncts that are not barbiturates, such as haloperidol, beta-adrenergic antagonists, and propofol [27, 33]. Barbiturates are a highly effective treatment for alcohol withdrawal and are considered first- or second-line treatment [13, 34]. As our first case highlights, phenobarbital can lead to significant improvement in symptoms in patients with benzodiazepine-resistant alcohol withdrawal. Dexmedetomidine as an adjunct to phenobarbital in critically ill patients with alcohol withdrawal has been associated with longer ICU and hospital length of stay, higher rates of intubation, higher rates of delirium, and a higher number of doses of post-load phenobarbital boluses compared to those not receiving dexmedetomidine [31]. 

The EvADE (Effect of Adjunctive Dexmedetomidine in the Treatment of Alcohol Withdrawal Compared to Benzodiazepine Symptom-Triggered Therapy in Critically Ill Patients) study examined the use of dexmedetomidine as an adjunct therapy for alcohol withdrawal patients admitted to the ICU and found that patients who received dexmedetomidine plus symptom-triggered therapy with benzodiazepines had an increased ICU length of stay, increased benzodiazepine requirements while in the ICU, and increased physical restraint use compared to the group that received symptom-triggered therapy alone [35]. The increased overall benzodiazepine requirements in the dexmedetomidine group were thought to be related to the fact that they had longer ICU length of stay. The use of physical restraints is concerning. We know physical restraints lead to patient harm and even death [36]. Furthermore, patients receiving physical restraints describe the experience as traumatic [37]. One can only assume that patients who are inadequately treated for their alcohol withdrawal and physically restrained would experience similar trauma. 

Our two patients are examples of how medications that fail to address the underlying pathophysiology may lead to detrimental patient experiences. Although our first patient appeared calm and sedated, his description of his mental anguish would make one pause before reaching for dexmedetomidine again as a sole agent in the management of alcohol withdrawal. Our second patient seized despite having normal vital signs while being administered dexmedetomidine, showing that dexmedetomidine is not effectively addressing the increased excitatory tone and decreased inhibitory tone responsible for alcohol withdrawal, thus resulting in this patient seizing. 

There is no convincing mechanism-based data to support the use of dexmedetomidine as a single therapy for alcohol withdrawal, and the current literature is divided on its utility as an adjunct therapy. Benzodiazepines not only treat the patient’s vital signs, but they also address the underlying etiology of alcohol withdrawal. Furthermore, there is a concern that alcohol withdrawal is associated with kindling [38]. Therefore, if the patient’s alcohol withdrawal is not treated effectively, the patient’s next presentation for alcohol withdrawal could be more severe.

## Conclusions

Alcohol withdrawal is a very common cause of hospital presentations. Clinicians are starting to propose “benzodiazepine sparing” protocols. We present two patients who had severe alcohol withdrawal and demonstrated the significant limitations of dexmedetomidine. We propose that benzodiazepine sparing alone is an inappropriate reason to utilize alcohol withdrawal protocols that use dexmedetomidine alone. We believe that there is currently not enough data to support the routine use of dexmedetomidine without sufficient concurrent GABA agonist (benzodiazepine and/or barbiturate) therapy. These two cases demonstrate the need for a well-designed randomized controlled trial where the outcome is patient-centered as opposed to one related to vital signs or a RASS score. Furthermore, it is also important to look at long-term outcomes to see if there is a change in later visits for alcohol withdrawal and overall risk for alcohol-related neurologic illness.
